# The influence of seismic frequency spectrum on the instability of loess slope

**DOI:** 10.1038/s41598-023-38016-w

**Published:** 2023-07-06

**Authors:** Chaoyu Chang, Feng Qiao, Jingshan Bo, Da Peng, Qi Li

**Affiliations:** 1 Institute of Disaster Prevention, Sanhe, 065201 China; 2 Hebei Key Laboratory of Earthquake Disaster Prevention and Risk Assessment, Sanhe, China; 3 Key Laboratory of Earthquake Engineering and Engineering Vibration, Institute of Engineering Mechanics, China Earthquake Administration, Harbin, 150080 China

**Keywords:** Natural hazards, Solid Earth sciences, Engineering

## Abstract

The input of seismic wave with different frequency has a significant impact on loess slope instability. On the basis of field investigation and experiments, the particle flow software PFC^2D^ was used to explore the effect of seismic frequency spectrum on slope instability through the process of calibrating soil microscopic parameters, model establishment, seismic wave input and other processes. The results show that: 1. The low-frequency component of the input wave is the main frequency band that causes the slope instability, the slope has amplifying effect on the low-frequency input wave, and the slope has a "filtering" effect on the high-frequency input wave; 2. The instability of the slope will cause an increase in frequency components above 10 Hz; 3. The special structure of the slope is one of the main reasons for the instability of the slope. This result has theoretical and practical significance for earthquake landslide prevention and monitoring and early warning.

## Introduction

The occurrence of seismic landslide is essentially the action of seismic waves on the slope, which causes the slope to change from a static state to a moving state^1^. The violent vibration causes the rock and soil structure to loosen and deform, and slip plane is penetrated^2,3^. Natural seismic waves contain many frequency bands^4^. Although seismic waves of any frequency will cause the slope to vibrate, when the frequency of the earthquake is close to or the same as the natural frequency of the slope, the seismic waves will be amplified several times or even dozens of times due to resonance, and the stress and displacement deformation of the slope will increase, the probability of a landslide will increase accordingly^5,6^.

Many practical investigations and theoretical studies of earthquake landslides show that the three elements (amplitude, frequency spectrum and duration) of seismic waves determines the degree of slope damage in the earthquake^7,8^.Since seismic frequency spectrum is complex, the study of seismic frequency spectrum on slope dynamic response is not sufficient, but facts and existing studies have shown that seismic waves with different frequency spectrum has significant influence on slope stability. Liu et al.^9^ confirmed that the seismic frequency spectrum have a certain influence on the slope stability by using the finite method; Xu et al.^10^ showed that the slope soil has an amplifying effect on the low-frequency part of the input seismic waves and a filtering effect on the high frequency part by useing FLAC3D. Zhang et al.^11^ analyzed the acceleration and displacement response of the slope model under different frequency spectrum seismic waves, and showed that when the input seismic wave characteristic period is greater than 0.65 s, the slope displacement response increases significantly, and the PGA amplification factor of the slope shows an increasing trend. Shi et al. ^12^ uses wavelet packet analysis to decompose the dynamic earth pressure time-domain curve based on large-scale shaking table experiments, confirming that low-frequency waves play a dominant role in the dynamic earth pressure response, and the slope has a filtering effect on the high frequency part of the seismic wave. Chang et al. ^13^ simulated the failure process of loess seismic landslide based on PFC, and confirmed that low-frequency vibration had a stronger destructive effect on landslide, and the high-frequency component of slope vibration increased after failure.

The occurrence of landslide is a process of rock and soil damage and movement with sliding, translation and rotation. Finite element, finite difference and other continuum mechanics methods have limitations. On the basis of field investigation and experiments, the particle flow software PFC2D was used to explore the effect of ground motion spectrum on slope instability through the process of calibrating soil microscopic parameters, model establishment, power input and other processes. The main frequency bands that cause slope instability are obtained by analysis.

## Calculation model

### Slope model

**Figure 1:**
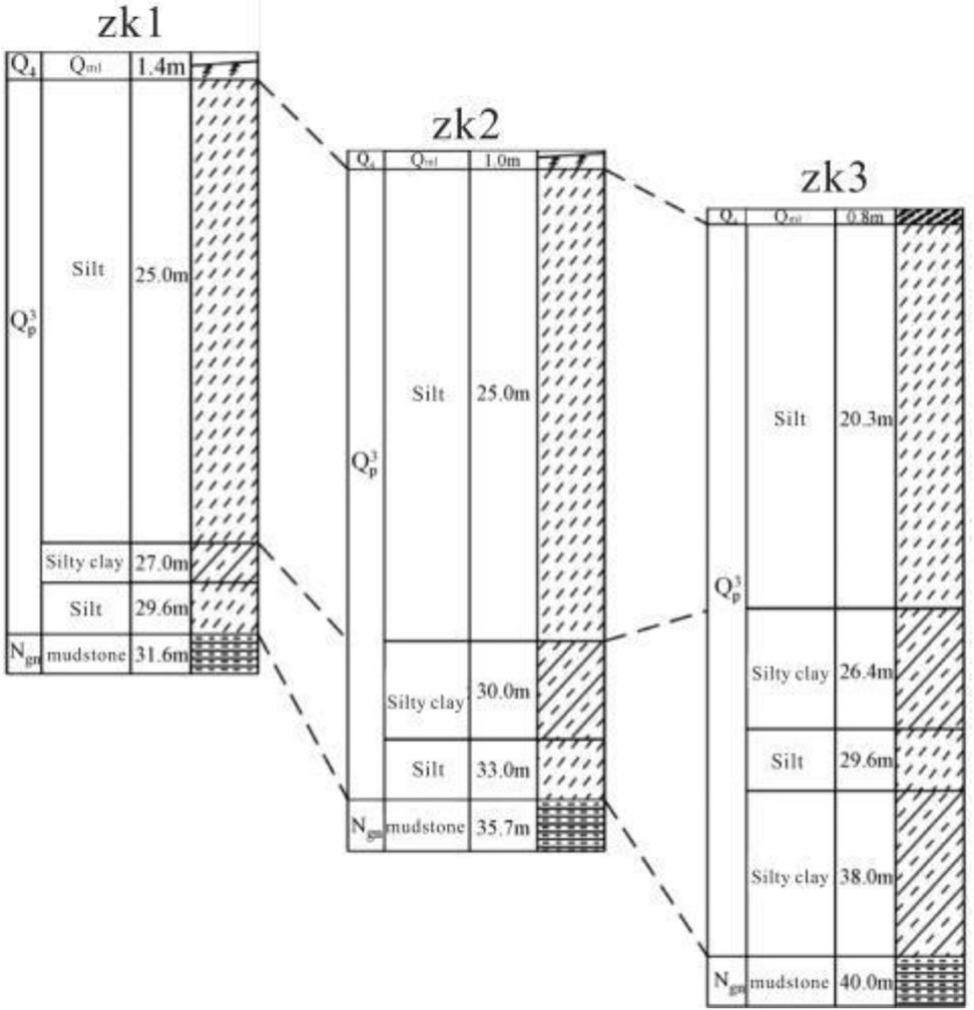
The stratums of Subao landslide based on the survey.

**Figure 2:**
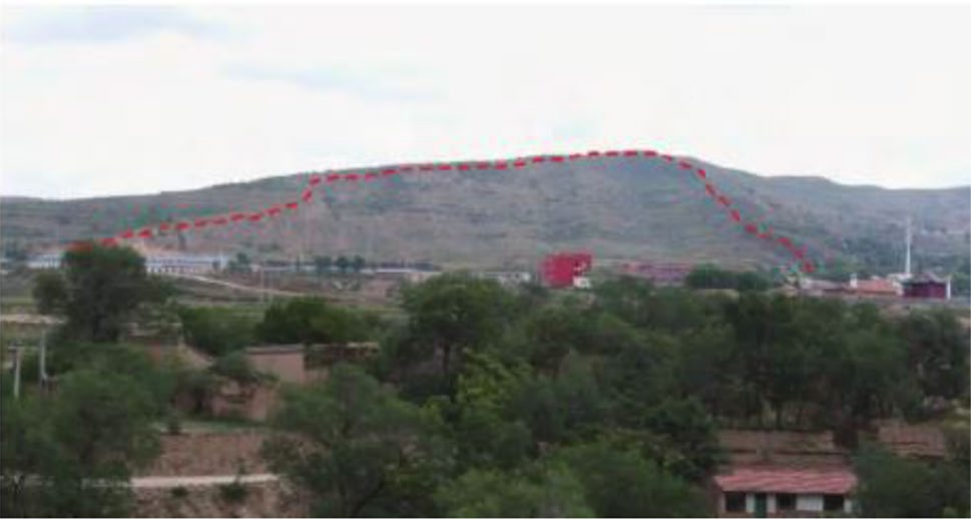
The overview of Subao landslide.

**Figure 3:**
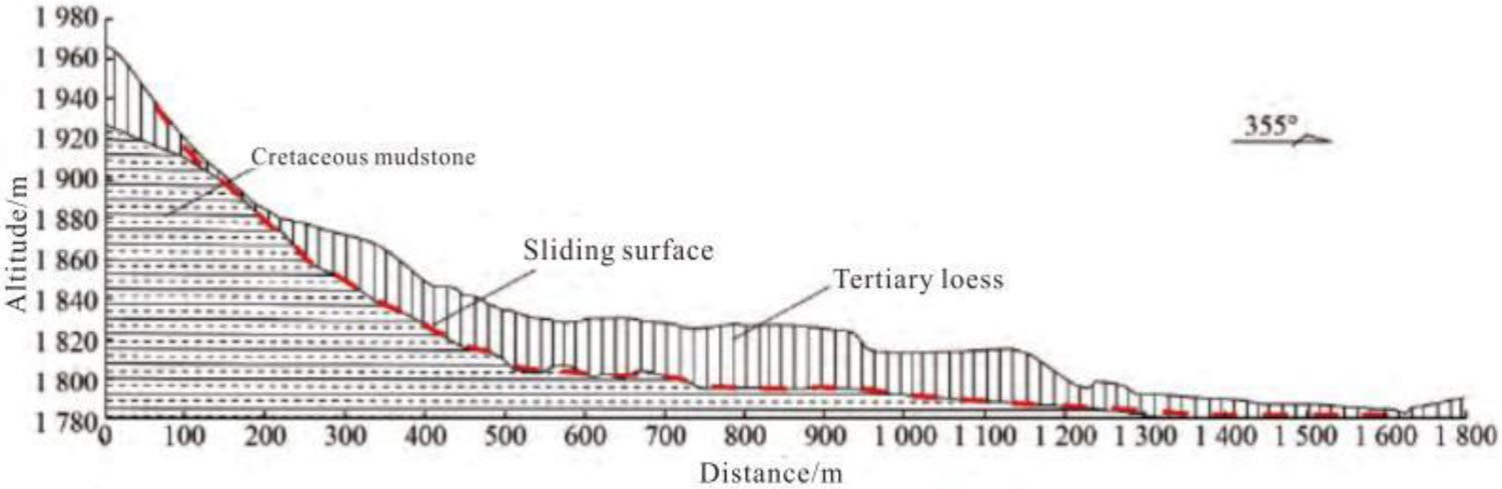
The stratigraphic profile of Subao landslide.

The Haiyuan 8.5 magnitude earthquake that occurred on December 16, 1920 induced hundreds of loess landslides^14,15^. Due to the special geographical location and climatic conditions, most of these loess earthquake landslides are still well-preserved^16^. The Subao landslide induced by the Haiyuan earthquake is selected as the calculation model of this paper. The Subao Landslide (105°30′43″E, 35°32′8″N) is located in Subao Village, Subao Township, Xiji County,China.The plane form is dustpan-like, wide at the front and narrow at the back. The main sliding direction is 335°. The landslide is 1300 m long and 550 m wide. According to field survey and geological exploration (Figs. 1, 2 and 3), the landslide is a loess-mudstone landslide: the upper part is the Late Pleistocene loess, and the lower part is the Tertiary red-brown mudstone^17^.

### Calculation parameters

**Figure 4:**
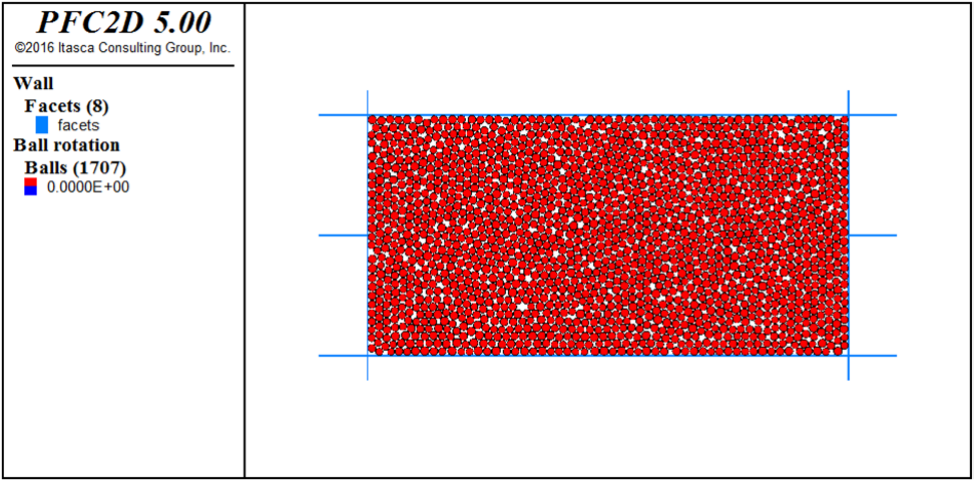
The direct shear test model for the identification of mesomechanical parameters.

**Figure 5:**
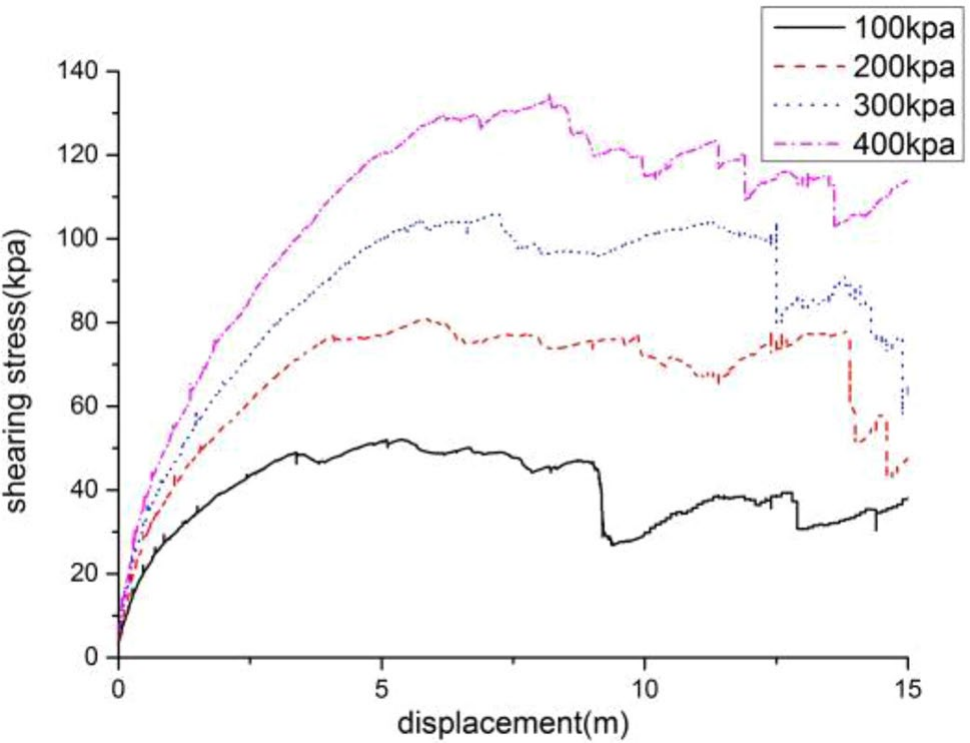
The shear force and displacement curve in the direct shear simulation tests.

**Figure 6:**
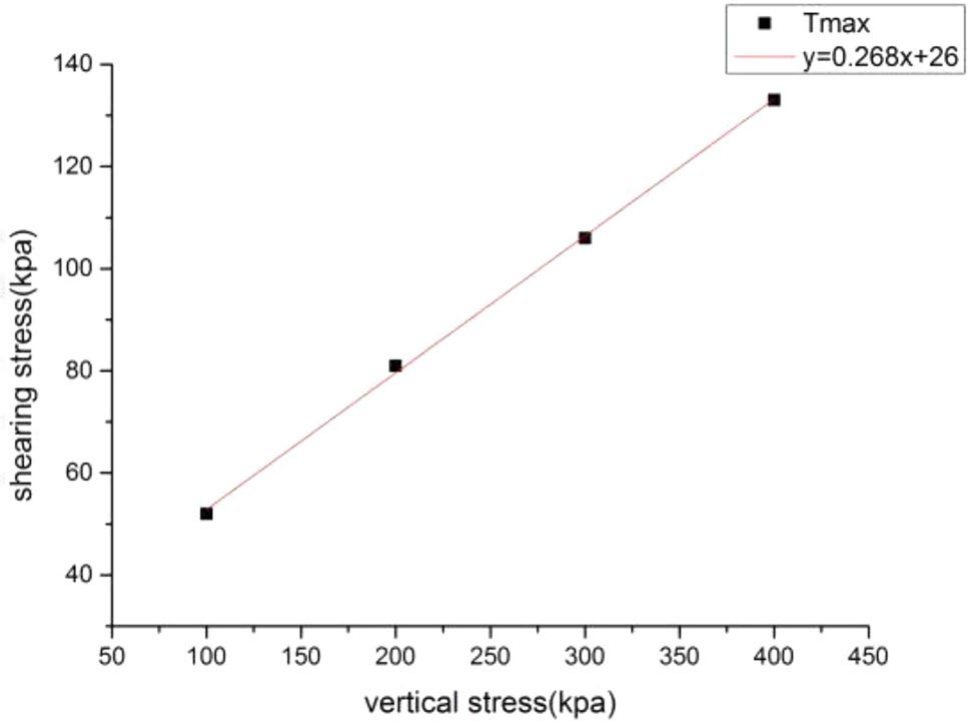
The simulated direct shear test strength curve.

The direct shear test model is used to calibrate the meso-mechanical parameters. The size of the model is 500 m × 400 m (Fig. 4), and the total number of particles generated is 1707. The particle-to-particle contact model selects the parallel bond (linearpond) model, and the particle meso-mechanical parameters of loess and mudstone are listed in Table 1. Taking loess as an example, under the conditions of meso-mechanical parameters in Table 1, vertical stresses of 100, 200, 300, and 400 kPa were applied to the direct shear model to obtain the shear stress-displacement relationship curve (Fig. 5). Then draw the direct shear test strength curve (Fig. 6), and calculate the macro-mechanical parameters of the material in the direct shear test. The macro-mechanical parameters of rock and soil obtained by numerical simulation of direct shear test. Cohesion c = 26 kPa and internal friction angle φ = 15°. By comparing with c = 21.3 ~ 28.7 kPa and φ = 11.2 ~ 18.3° obtained by indoor physical test, it can be seen that the mechanical parameters obtained by numerical simulation are within its range, and the meso-parameters are available.

**Table 1:** The particle mechanical parameters in models.

	Particle density (kg·m^−3^)	Particle diameter (m)	Contact stiffness (pa)		Boding strength (pa)		Friction coefficient	Damping coefficient
			Normal direction	Shear direction	Normal direction	Shear direction		
Loess	2000	1 ~ 2.0	$1 \times 10^{8}$	$2 \times 10^{4}$	$1 \times 10^{8}$	$2 \times 10^{4}$	0.6	0.7
Mudstone	2400	1 ~ 2.0	$5 \times 10^{8}$	$5 \times 10^{4}$	$5 \times 10^{8}$	$5 \times 10^{4}$	0.7	0.7

### Calculation model

**Figure 7:**
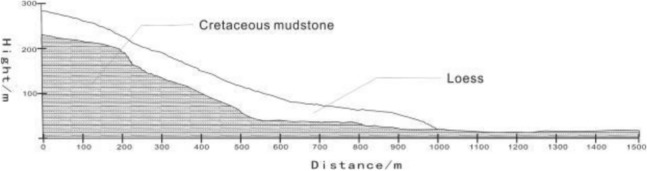
The strata profile after the landscape restoration.

**Figure 8:**
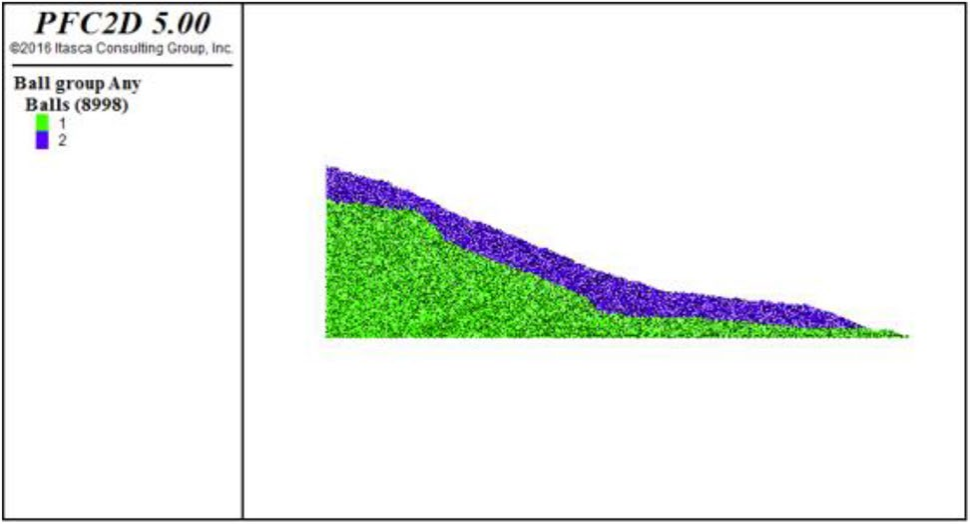
The slope model in PFC.

According to the geological section, the landslide topography of the Subaocun landslide was restored by using the surface fitting program in MATLAB^18^. The restored stratigraphic section is shown in Fig. 7. The wall-ball method is used in PFC^2D^ to establish the original landslide model. The specific steps are as follows: ①Create a rectangular area larger than the column profile in PFC2D and generate particles; ②Import the landslide profile established in AutoCAD, and group the particles according to the landslide boundary ③According to the meso-mechanical parameters obtained by parameter calibration, the particles are assigned; ④Gravity is applied to make the landslide reach the initial stress equilibrium state under the action of gravity; ⑤The particles outside the section area are deleted; ⑥Steps 4 and 5 are repeated until the particles are complete Reconcile with the profile view to achieve a state of stress balance. According to the above steps, the PFC2D model of the Subao landslide is obtained, as shown in Fig. 8. The model is 1500 m long, 230 m high, and has 8998 particles. Under the action of gravity, the slope is stable.

## Seismic waves input

**Figure 9:**
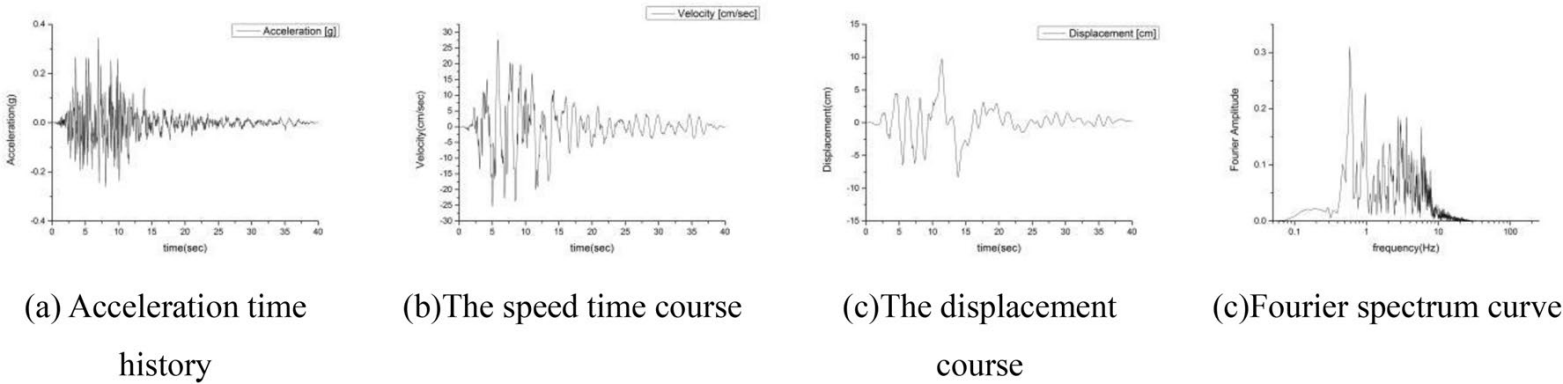
Seismic wave of Kobe.

**Figure 10:**
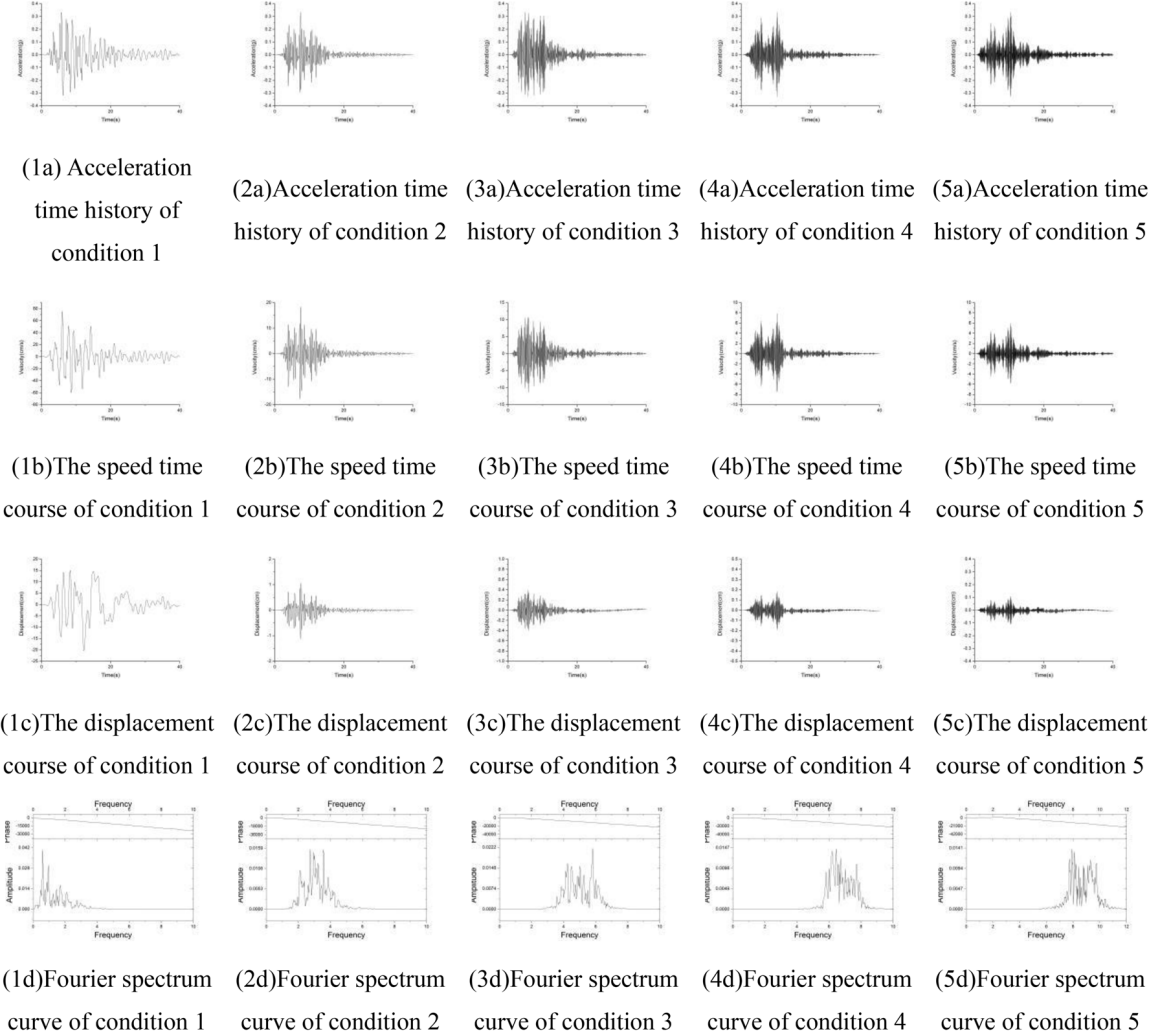
Seismic wave inputs.

In order to explore the influence of the seismic frequency spectrum on the instability of the loess slope, the Kobe seismic wave (NS direction) recorded by the Hanshin earthquake is selected as the original seismic data. The peak acceleration of the Kobe wave is 0.3347 g. Time history, displacement time history and the Fourier spectrum are shown in the Fig. 9. It can be clearly seen from the figure that the main frequency of the kobe wave is concentrated in 0–10 Hz, and the Butterworth filter is used for band-pass filtering, and the interception frequencies are respectively selected as 0–2 Hz, 2–4 Hz, 4–6 Hz, 6–8 Hz and 8–10 Hz, Adjust the amplitude of the five acceleration time courses after filtering, so that the five acceleration peaks are all 0.3347 g, and the adjusted acceleration time history, velocity, displacement and acceleration Fourier spectrum are shown in the Fig. 10. Use the 5 acceleration time histories of different frequency bands as input waves, corresponding to conditions 1–5 (Table 2).

**Table 2:** 5 conditions of seismic wave input.

Condition number	1	2	3	4	5
Peak acceleration	0.3347 g	0.3347 g	0.3347 g	0.3347 g	0.3347 g
excellent frequencies	0–2 Hz	2–4 Hz	4–6 Hz	6–8 Hz	8–10 Hz

## Results

**Figure 11:**
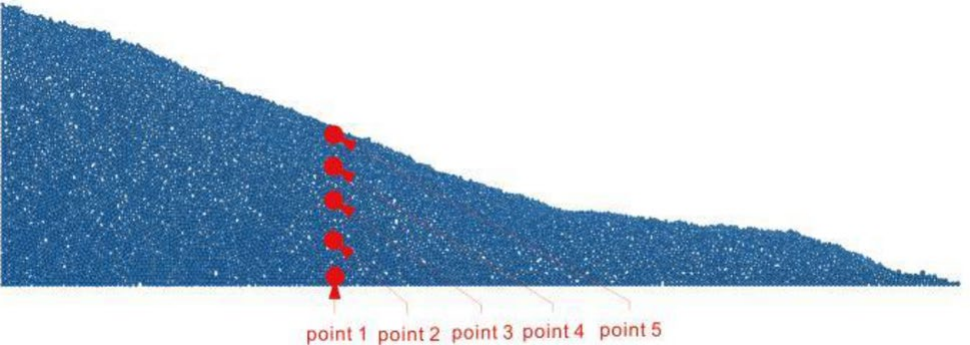
The measure locations.

In order to monitor the acceleration, velocity, displacement and spectrum change characteristics of the slope under the dynamic action of different frequency spectrums, the movement of particles of different heights at 10 m, 50 m, 90 m, 130 m and 170 m within the slope were monitored. The location is shown in Fig. 11.

### Acceleration response

**Figure 12:**
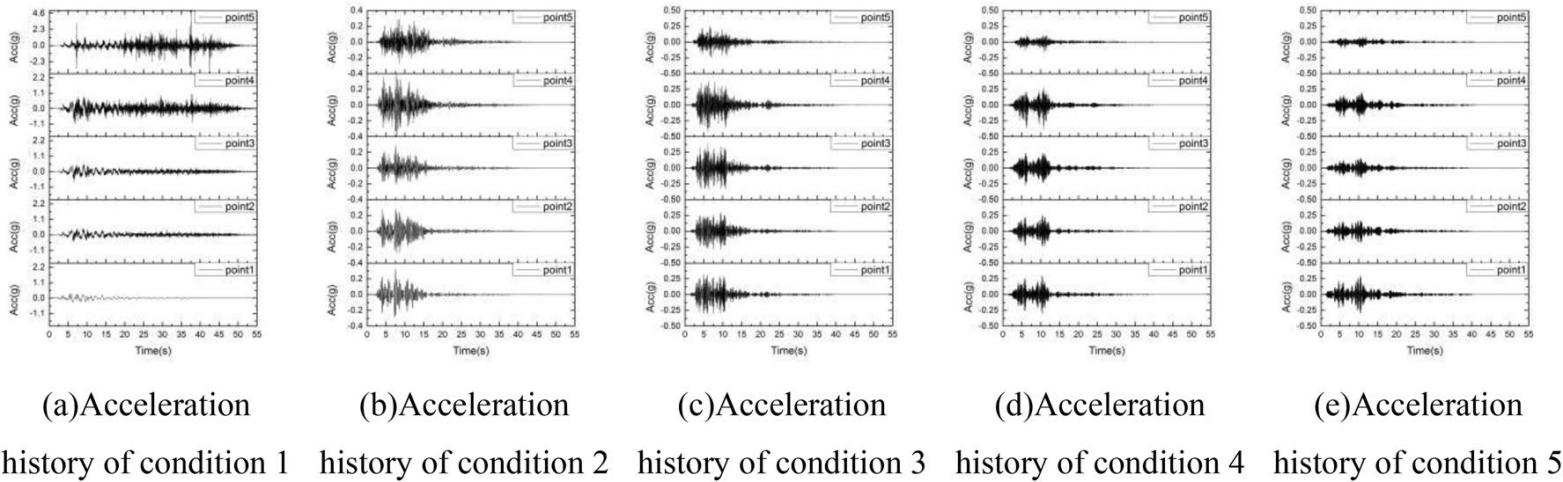
The acceleration history of the sphere at 5 positions under 5 conditions.

By monitoring the calculation process of the slope model, the acceleration curve of the calculation process of the sphere at 5 positions under 5 conditions is obtained, as shown in the Fig. 12. It can be seen from the figure that under different frequency acceleration inputs, the acceleration changes from bottom to slope surface are very different. Under the power input with an excellent frequency of 0–2 Hz, the peak acceleration amplification increases from bottom to surface. 20 s After the seismic wave input, the acceleration of monitoring point 4 and 5 changes drastically, which is the key to instability. Under the input with excellent frequencies of 2–4 Hz and 4–6 Hz, the amplitude of the peak acceleration from bottom to surface does not change much. Under the input with excellent frequencies of 6–8 Hz and 8–10 Hz, the peak acceleration becomes smaller on the monitoring point 5 which near the slope position. The results show that the frequency spectrum of seismic wave has a great influence on the dynamic response of the slope.

**Figure 13:**
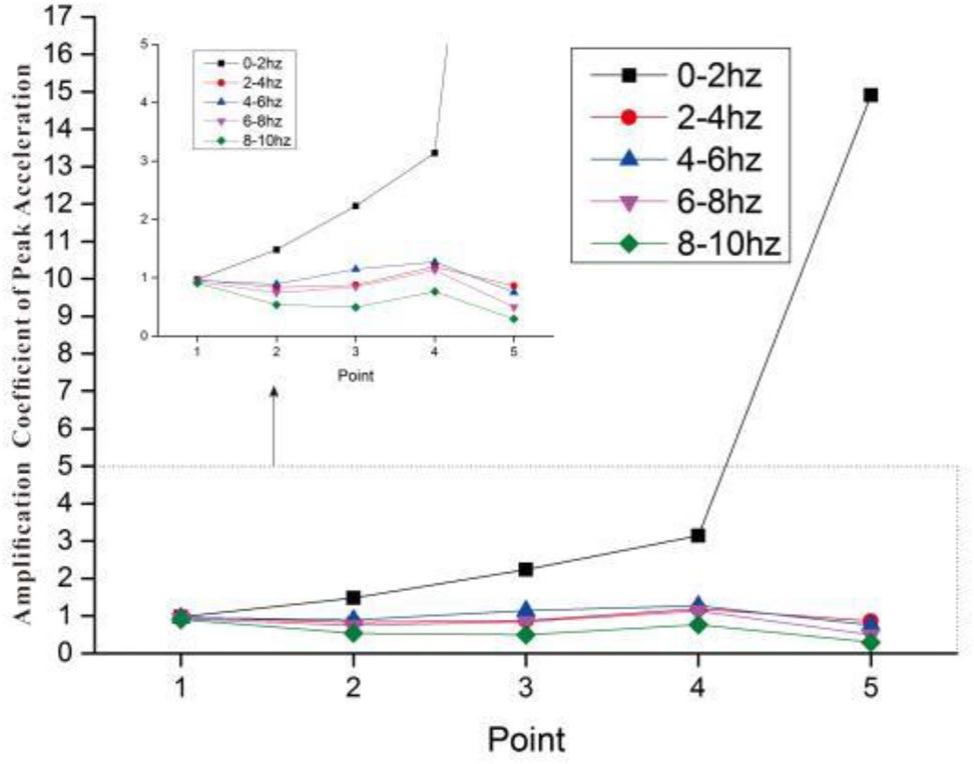
The amplification coefficient of peak acceleration under 5 conditions.

In order to further illustrate the influence of the seismic frequency spectrum on the peak acceleration, the peak acceleration magnification under different frequency seismic waves input is plotted in the Fig. 13. It can be clearly seen from the figure that the magnifications of monitoring point 1 are all close to 1, which indicated the dynamic boundary conditions are reasonable. Under the input with the excellent frequency of 0–2 Hz, the amplification of monitoring point 5 is the largest, which can reach 17.9. Under the input with the excellent frequency of 2–4 Hz, 4–6 Hz, 6–8 Hz and 8–10 Hz, The amplifications of the monitoring point 5 are all less than 1, maintaining a stable state. The magnification of monitoring point 4 is larger than that of other points, which is related to its location where is the interface.

### Spectrum

**Figure 14:**
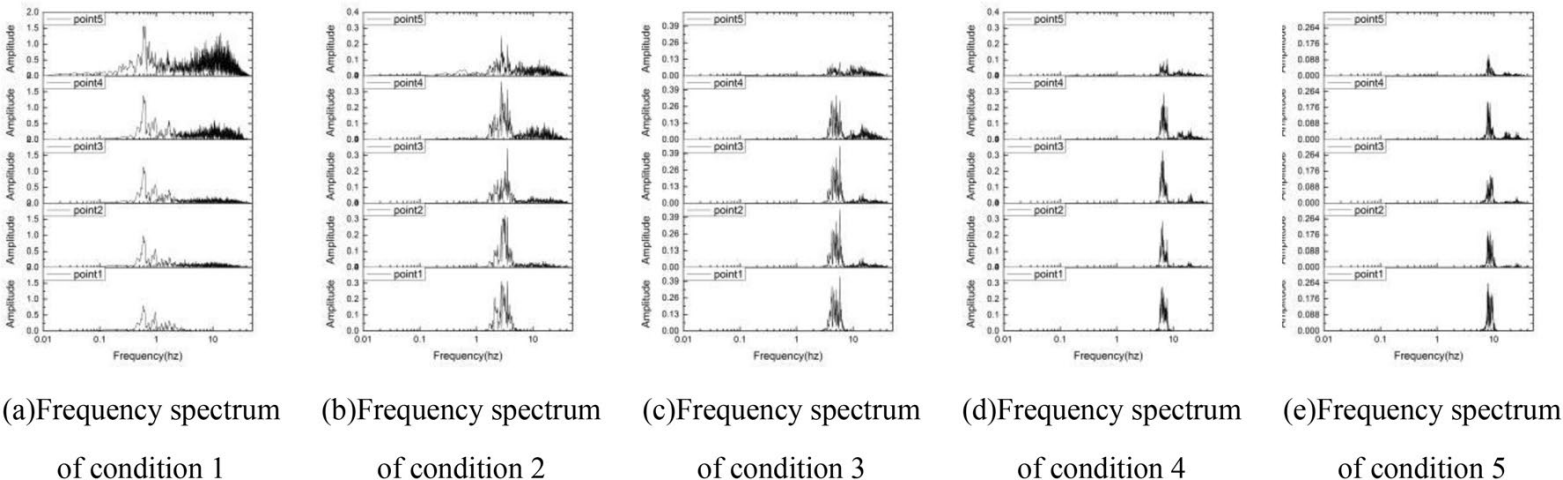
The frequency spectrum of the sphere at 5 positions under 5 conditions.

In order to reveal the influence of the frequency spectrum on the loess slope, the Fourier spectrum of the acceleration at the 5 detection points under 5 inputs is calculated and shown in the Fig. 14. It can be seen from the figure that the Fourier spectra of the five detection points have all major changes from bottom to surface no matter what frequency the input is, showing the unique spectral characteristics of the slope itself, the closer to the slope. The closer to the slope surface, the more high-frequency components. The smaller the input frequency, the larger the Fourier amplitude spectrum of the high-frequency components of the slope, which indicates that the collision frequency of particles in the slope increases, the acceleration changes more drastically, and the instability failure occurs. The frequency of the input power is different, and the amplification of the same frequency range at different locations is different. Under the input of the excellent frequency of 0–2 Hz, the part of the Fourier spectrum at 0–2 Hz gradually enlarges with the distance from the bottom surface. Under the power input with an excellent frequency of 8–10 Hz, the part of the Fourier spectrum at 8–10 Hz shows a decreasing trend with the distance from the bottom surface, which further shows that the input wave frequency spectrum has a significant influence on the instability and damage of the loess slope.

### Velocity

**Figure 15:**
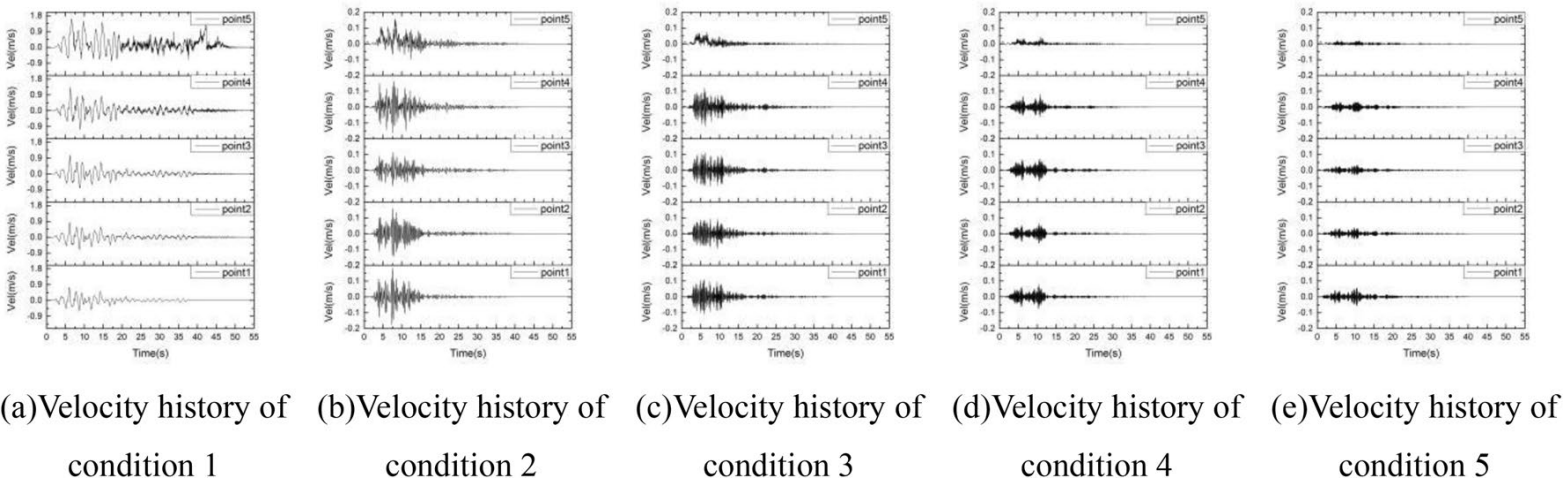
The velocity history of the sphere at 5 positions under 5 conditions.

The velocity history curves of the sphere at 5 positions under 5 conditions is shown in the Fig. 15. It can be seen from the figure that under the input with an excellent frequency of 0–2 Hz, the closer the monitoring point is to the slope surface, the more the velocity changes. Before 20 s, the velocity trends of the 5 monitoring points are relatively consistent. After 20 s, the velocity waveforms are quite different. The monitoring points 4 and 5 near the slope surface still have high velocities, but near the bottom, the velocities of monitoring point 1 and 2 are close to zero. Under the power inputs with excellent frequencies of 2–4 Hz, 4–6 Hz, 6–8 Hz and 8–10 Hz, the trend of the velocity curves of monitoring points 1–4 is consistent, and the velocity of monitoring point 5 appears more "positive" than “negatie”, and these points and zero axis form a larger area, which is the performance of the displacement caused by the instability of the slope particles. Under the input with excellent frequencies of 4–6 Hz, 6–8 Hz and 8–10 Hz, the speed amplitude of monitoring point 5 is the smallest value among the 5 monitoring points, indicating that under the action of soil damping, much energy is consumed, which lead the destructive power weak.

### Displacement

**Figure 16:**
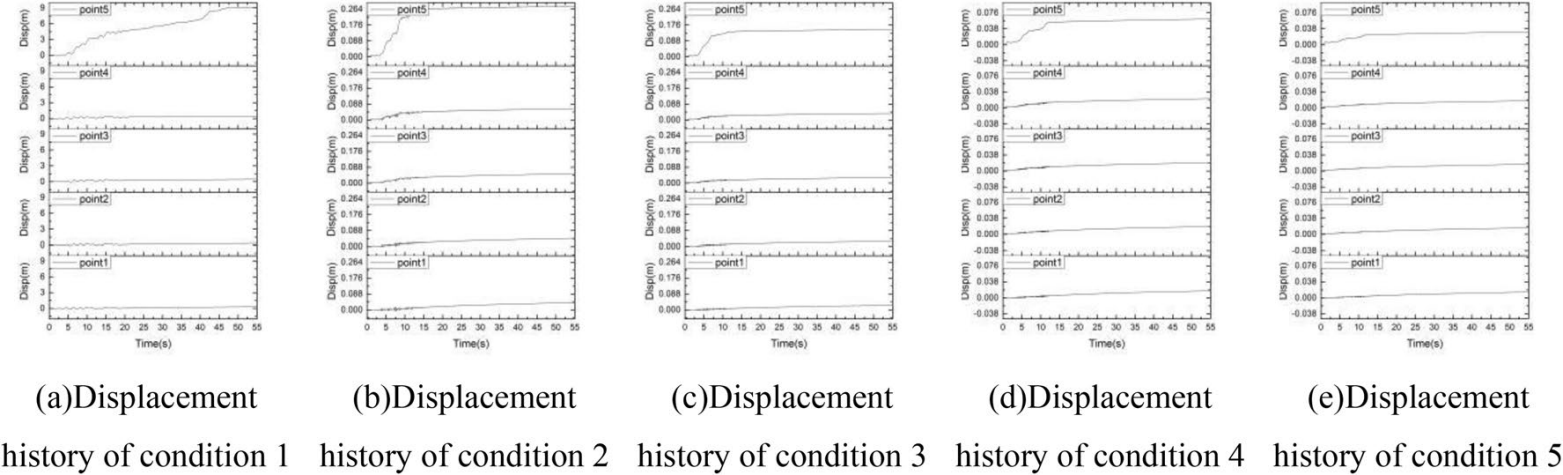
The displacement history of the sphere at 5 positions under 5 conditions.

The displacement history curve of the sphere at 5 positions under 5 conditions is shown in the Fig. 16. It can be seen from the figure that under the input with an excellent frequency of 0–2 Hz, the displacement of the monitoring point 5 continues to increase from 5 s, and stabilizes after 45 s. The maximum displacement reaches 9 m. Under the input with excellent frequencies of 2–4 Hz, 4–6 Hz, 6–8 Hz and 8–10 Hz, the displacement of monitoring point 5 has a certain increase in 5–15 s, but the change range is not large, and the maximum value is 0.26 m under the power input of 2–4 Hz. No matter what kind of input, the displacement changes of monitoring points 1–4 are small, and are always near 0 point, indicating that there is no instability damage occurred.

**Figure 17:**
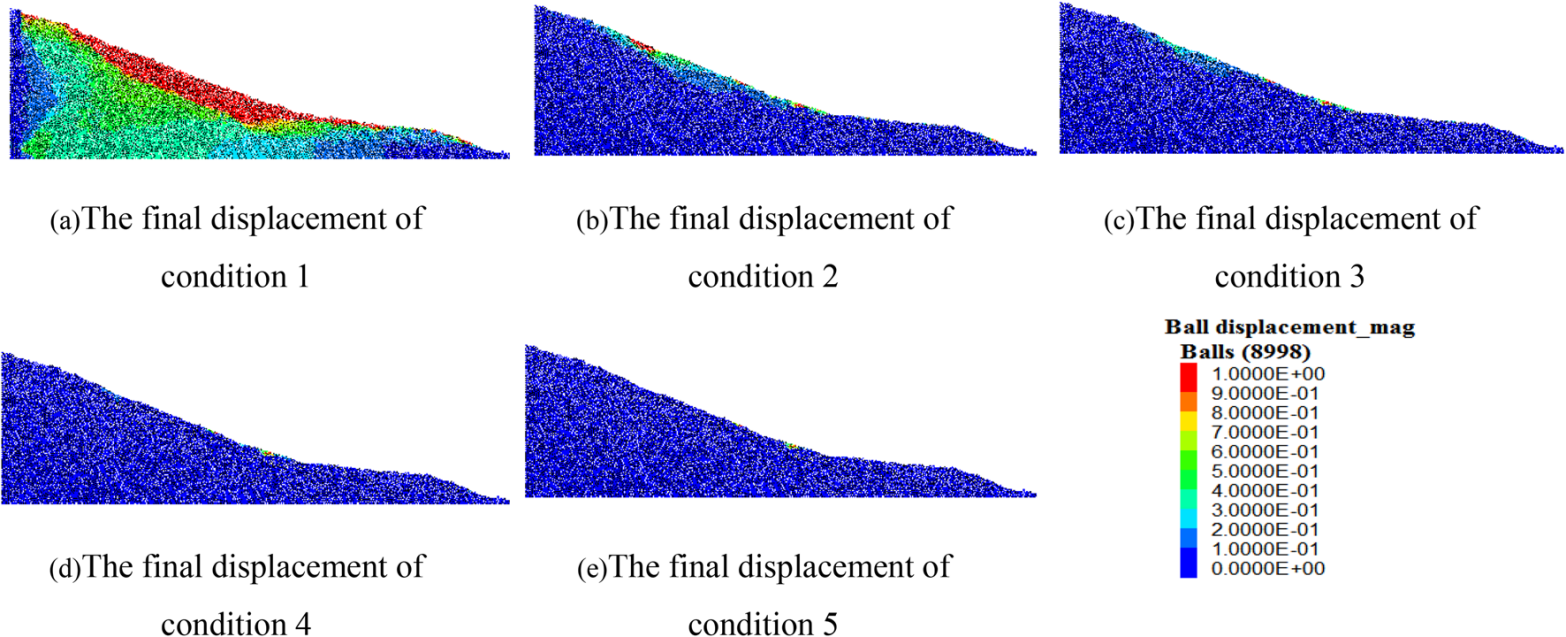
The final displacement of the slope.

In order to further compare the displacement of the slope under the 5 kinds of input, the final displacement of the slope is shown in the Fig. 17. It can be clearly seen from the figure that under the input with an excellent frequency of 0–2 Hz, the slope is obviously unstable. The upper loess and the lower mudstone have an obvious displacement difference, and the sliding surface mainly relies on the stratum interface, and the overall shape is irregular arc. As the frequency of input power increases, the degree of slope instability becomes smaller. Under the input of excellent frequency of 2–4 Hz, there is only instability in part of the slope surface, and it is concentrated at the toe and shoulder, where are usually the concentrated areas of tensile failure and compression failure. Under the input with excellent frequencies of 4–6 Hz, 6–8 Hz and 8–10 Hz, only a small range of instability occurs at the toe of the slope, which has no obvious impact on the slope as a whole.

## Conclusion

In order to explore the impact of seismic frequency spectrum on slope instability, on the basis of field investigation and experiments, the particle flow software PFC^2D^ was used to explore the effect of ground motion spectrum on slope instability through the process of calibrating soil microscopic parameters, model establishment, power input and other processes, and obtained the following conclusions through analysis:

1. The frequency spectrum characteristics of seismic wave have a significant impact on the instability of loess slopes. The low-frequency components (0–2 Hz) in the input seismic wave are the main frequency bands that cause slope instability and destruction. Under low-frequency input, the peak acceleration, acceleration amplification factor, speed and displacement near the slope surface are relatively large, and obvious instability and damage phenomena occur; under high-frequency input, the peak acceleration, acceleration amplification factor, speed and displacement near the slope surface are all small, and the slope has a "filtering" effect on the input wave.
2. The destruction of the slope will cause an increase in frequency components above 10 Hz. Regardless of the input seismic wave in any frequency band, under the dual influences of the earthquake and the empty surface, the collision between the broken soil and the original slope is intensified, which further causes the instability and damage of the slope. Due to the “filtering” effect of the slope on the high-frequency components of seismic wave and the increase in high-frequency components caused by rupture, the high-frequency components of seismic wave recorded on slopes mostly reflect the damage of the slope. This conclusion can be used for monitoring and early warning of earthquake landslides.
3. The special structure of the slope is one of the main reasons for the instability of the slope. Due to the sudden change of soil properties and wave impedance, the acceleration and velocity increase at the contact surface of mudstone and loess, which is easy to lose stability at the junction and form a sliding surface. Regardless of the frequency of seismic wave input, the toe of the slope is the first part to lose stability. In the prevention of earthquake landslide disasters, the reinforcement treatment of slope contact surface and slope toe requires attention.

It is worth pointing out that the particle flow method in this paper does not consider the structural properties of soil and the effects of groundwater in the simulation. According to the experience of field investigations and laboratory tests, the structural properties and water sensitivity of loess play an important role in slope instability, which should be taken into consideration in the numerical simulation. The conclusion of this article is the regularity of seismic frequency spectrum on the instability of loess slopes. In future research, the structural and water sensitivity of the soil should be fully considered to quantify the impact of ground motion frequency spectrum on the instability of slopes.

## Data Availability

The datasets used and/or analysed during the current study available from the corresponding author on reasonable request.
